# Resveratrol: Molecular Mechanisms, Health Benefits, and Potential Adverse Effects

**DOI:** 10.1002/mco2.70252

**Published:** 2025-06-11

**Authors:** Zhuo‐qun Ren, Sheng‐yuan Zheng, Zhengcheng Sun, Yan Luo, Yu‐tong Wang, Ping Yi, Yu‐sheng Li, Cheng Huang, Wen‐feng Xiao

**Affiliations:** ^1^ Department of Orthopedics Xiangya Hospital Central South University Changsha Hunan China; ^2^ National Clinical Research Center for Geriatric Disorders Xiangya Hospital Central South University Changsha Hunan China; ^3^ Department of Clinical Medicine Xiangya Medicine School, Central South University Changsha Hunan China; ^4^ Department of Orthopaedic Surgery China‐Japan Friendship Hospital Beijing China

**Keywords:** aging, cancer, pathogenesis, potential adverse effects, resveratrol, therapeutic mechanisms

## Abstract

Resveratrol (RES), a naturally occurring polyphenolic compound, has garnered significant attention due to its diverse biological activities, which include anti‐inflammatory, antioxidant, and antiaging properties. This review synthesizes current evidence concerning the molecular mechanisms, therapeutic efficacy, and safety profile of RES across a variety of pathologies, with an emphasis on the latest research conducted in recent years. Mechanistically, RES is known to modulate critical signaling pathways such as the activation of sirtuin 1. These actions collectively contribute to the attenuation of oxidative stress, regulation of apoptosis, and promotion of autophagy. Preclinical studies have demonstrated the potential of RES in the mitigation of degenerative musculoskeletal disorders, cardiovascular diseases, cancer progression, and neurological diseases. Given the low bioavailability of RES and the potential for adverse reactions in clinical applications, we summarize and discuss its safety profile while outlining future research directions. This review underscores the therapeutic versatility of RES while advocating for rigorous pharmacokinetic optimization, standardized dosing protocols, and large‐scale randomized controlled trials to validate its efficacy and safety in human populations.

## Graphical abstract

As a polyphenol substance with important potential, resveratrol has important potential in degenerative diseases, cardiovascular diseases, cancer, neurological diseases, etc. However, it is still necessary to pay attention to its bioavailability and potential adverse effects.

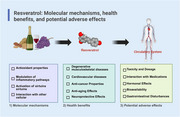

## 1 Introduction

Resveratrol (RES) (3,5,4′‐trihydroxystilbene) is a natural polyphenolic phytoalexin that exists in two geometric isomers: cis and trans. The trans‐isomer is noted for its superior stability and bioactivity, attributed to the minimized steric hindrance of its side chains [1, 2]. Although RES was first isolated by Takaoka in 1940 from white hellebore [3], it has been utilized for centuries in traditional Chinese medicine as “li lu” [4] and in India's Ayurvedic medicine [5]. It was not until 1992 that RES attracted considerable scientific interest, largely owing to the “French paradox.” This paradox highlights the unusually low incidence of cardiovascular mortality in the French population, despite a diet rich in saturated fats, potentially attributed to their significant red wine consumption [6]. RES is found in over seventy plant species, with notable sources including grapes, peanuts, cacao, and various Vaccinium species. Among these, grape‐derived products, particularly red wine, are the primary dietary contributors to RES intake [7].

RES has been thoroughly investigated for its multifaceted effects, including anti‐inflammatory, antiaging, anticancer, and antioxidant properties, along with its potential therapeutic benefits in neurodegenerative disorders, degenerative musculoskeletal diseases, and both cerebrovascular and cardiovascular diseases (CVDs) [8]. Despite these promising effects, challenges persist, particularly regarding the low bioavailability and high bioactivity of RES, a phenomenon commonly referred to as the “RES paradox” [9]. Moreover, the potential adverse effects of RES in clinical applications necessitate further investigation to fully assess its safety and efficacy.

This review underscores the health benefits of RES, exploring the molecular mechanisms and cellular targets involved, with particular emphasis on its positive effects on degenerative musculoskeletal diseases, cardiovascular health, neuroprotection, anticancer properties, and antiaging effects. Additionally, it delves into the potential adverse effects of RES, including concerns related to dosage, toxicity, hormonal influences, and interactions with other medications in clinical settings. Furthermore, we summarize recent advancements in research aimed at enhancing the bioavailability of RES, providing valuable insights for the innovation of its clinical applications.

## 2 Molecular Mechanism of RES

**FIGURE 1:**
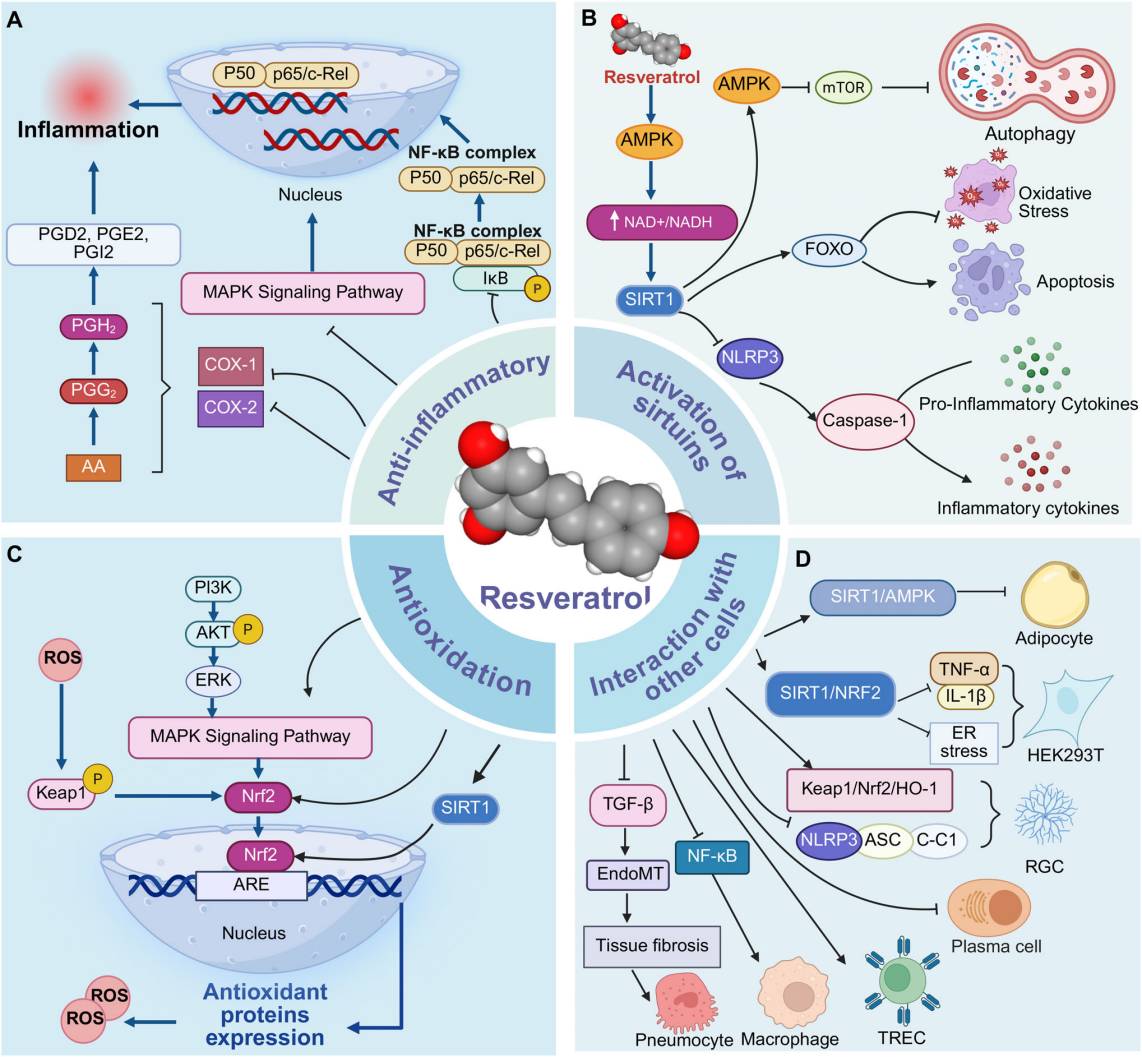
Structure and molecular mechanism of RES. The molecular mechanisms underlying the effects of RES primarily involve its antioxidant properties, anti‐inflammatory actions, activation of sirtuins, and interactions with various cellular pathways. AA, arachidonic acid; AMPK, AMP‐activated protein kinase; ASC, apoptosis‐associated speck‐like protein containing a CARD; ARE, antioxidant response element; AKT, protein kinase B; C‐C1, caspase recruitment domain‐containing protein 1; COX, cyclooxygenase; ERK, extracellular signal‐regulated kinase; FOXO, Forkhead Box O; HO‐1, heme oxygenase‐1; IL‐1β, interleukin‐1β; MAPK, mitogen‐activated protein kinase; mTOR, mammalian target of rapamycin; NADPH, nicotinamide adenine dinucleotide phosphate; NF‐kB, nuclear factor‐kappa B; NLRP3, NACHT, LRR and PYD domains‐containing protein 3; Nrf2, nuclear factor erythroid 2‐related factor 2; P50, NF‐κB p50; P65, NF‐κB p65; PGE2, prostaglandin E2; PGD2, prostaglandin D2; PGI2, prostacyclin; PGH2, prostaglandin H2; PGG2, prostaglandin G2; PI3K, phosphoinositide 3‐kinase; ROS, reactive oxygen species; TGF‐β, transforming growth factor‐β.

RES has been demonstrated to influence a variety of cell‐signaling pathways and physiological systems, yielding anti‐inflammatory and antioxidant benefits. The central mechanism linking these effects appears to be sirtuins, a family of nicotinamide adenine dinucleotide (NAD+)‐dependent deacetylases [10]. Various sirtuin isoforms, with SIRT1 as the primary active molecule, exert distinct effects under a range of physiopathological conditions [11, 12, 13] (Figure 1).

### 2.1 Antioxidant Properties

Appropriate doses of RES appear to play a role in promoting the healing of various oxidative stress injuries. RES is implicated in oxidative stress through the p62–Keap1/Nrf2 [14, 15] and AKT/MAPK/Nrf2 signaling pathways [16, 17]. The specific benefits include upregulating the expression of antioxidant genes such as superoxide dismutase‐1 (SOD‐1) and catalase (CAT), enhancing the function of antioxidant enzymes like glutathione peroxidase [17] and heme oxygenase 1 [18], and inhibiting pro‐oxidants such as the caspase‐3 enzyme [19]. Notably, the SIRT1–NRF1/NRF2 pathway appears to serve as the upstream signaling mechanism through which RES exerts its protective effects against oxidative stress.

### 2.2 Modulation of Inflammatory Pathways

RES exerts its anti‐inflammatory effects through multiple signaling pathways, with the inhibition of the arachidonic acid (AA) pathway playing a crucial role [20]. RES can selectively decrease cyclooxygenase‐1 (COX‐1) activity and the hydroperoxidase activity of its isoenzyme, directly inhibit COX‐2 activity, and suppress the production of prostaglandins (such as PGD2, PGE2, PGI2) through the ERK1/2 and PI3K/AKT signaling pathways [21, 22, 23]. Furthermore, RES inhibits NF‐κB activation in a dose‐ and time‐dependent manner through multiple mechanisms [24], including SIRT1 activation, reduction of tumor necrosis factor (TNF) production [25, 26], and blocking the phosphorylation of p65 and IκB proteins [27]. In the MAPK pathway, RES inhibits the inflammatory response by suppressing the activation of ERKs and p38 MAPK pathways induced by phorbol 12‐myristate 13‐acetate (a tumor promoter) [28, 29]. In conclusion, RES mitigates inflammation through multiple signaling pathways, including the AA, NF‐κB, and MAPK pathways.

### 2.3 Activation of Sirtuins

Sirtuins are a family of NAD⁺‐dependent deacetylases classified as class III histone deacetylases, comprising seven distinct isoforms in mammals (SIRT1–SIRT7) [30]. Among these isoforms, SIRT1, SIRT6, and SIRT7 are localized in the nucleus and nucleolus, respectively; SIRT2 is predominantly cytoplasmic, while SIRT3, SIRT4, and SIRT5 are situated within the mitochondria [30, 31]. Evidence indicates that silencing sirtuins shortens lifespan, and these proteins appear to mediate the health‐promoting effects of caloric restriction and exercise, primarily through their upregulation by specific activators [32]. RES is recognized as the most potent polyphenolic activator of sirtuins in vitro. RES orchestrates the regulation of several critical signaling pathways through the activation of sirtuins, particularly SIRT1. This activation is linked to enhanced mitochondrial biogenesis and reduced oxidative stress and inflammation via the AMP‐activated kinase (AMPK)/PGC‐1α pathway [33, 34]. Moreover, RES can attenuate inflammation by deacetylating the p65 subunit of NF‐κB, thereby suppressing its transcriptional activity [35, 36]. It also regulates the SIRT1/NLRP3 pathway to prevent the assembly and activation of the inflammasome [37]. Furthermore, RES impacts the SIRT1/mTOR pathway by inhibiting mTOR activity, promoting autophagy, and facilitating the removal of damaged organelles and proteins, thereby maintaining cellular homeostasis [38, 39, 40]. Through the sirtuin/FOXO pathway, RES enhances the expression of antioxidant enzymes such as SOD and CAT, thereby reducing oxidative stress and safeguarding cells from damage [41, 42]. In conclusion, RES functions as an upstream signaling molecule with profound effects on the expression and activity of all sirtuin isoforms. As one of the primary SIRT1 activators, RES predominantly influences proteins modulated by fat and carbohydrate intake, suggesting that its effects may be particularly pronounced in individuals with a high‐fat diet. The pathways activated by RES hold significant therapeutic potential for a range of diseases.

### 2.4 Interaction with Other Cellular Pathways

As a nutritional or dietary supplement, RES interacts with various cell types. RES can act on adipocytes, increasing the expression of browning‐related genes in white adipose tissue through the SIRT1/AMPK pathway, thereby reducing fat accumulation [43]. Endoplasmic reticulum stress is a cellular response triggered when the protein folding capacity of the endoplasmic reticulum is overwhelmed, leading to the accumulation of misfolded or unfolded proteins. This accumulation contributes to the development of degenerative diseases [44].

RES can mitigate ovarian aging by enhancing the SIRT1/nuclear factor erythroid 2‐related factor 2 (NRF2) pathway, thereby regulating the expression of inflammatory and endoplasmic reticulum stress markers [45]. RES glycoside could alleviate cognitive dysfunction induced by lipopolysaccharide (LPS)‐induced sepsis‐associated encephalopathy, primarily by inhibiting endoplasmic reticulum stress and preserving the homeostasis of endoplasmic reticulum function in microglia [46]. In autoimmune diseases, RES can mitigate disease progression by inhibiting the NF‐κB pathway in macrophages, enhancing the function of regulatory T cells (Tregs), and reducing the production of autoantibodies in B lymphocytes [47]. RES can effectively ameliorate liver injury by targeting hepatocytes, restoring the shape and size of liver microvilli, and normalizing both the number and viability of mitochondria [48]. This effect may be attributed to RES's ability to reduce protein synthesis and alleviate the metabolic burden in the liver [49]. RES inhibits fibrogenic events such TGF‐β‐mediated cell signaling and endothelial‐to‐mesenchymal transition (EMT), which contribute to its antifibrotic abilities in reducing pulmonary fibrosis [50]. RES ameliorates retinal ischemia–reperfusion (I/R) injury by modulating the NLRP3 inflammasome and the Keap1/Nrf2/HO‐1 signaling pathway, thereby offering potential therapeutic benefits for glaucoma [51].

In summary, inflammation and oxidative stress are key factors in the development and progression of numerous degenerative diseases, and RES is likely to slow the progression of these conditions through the molecular mechanisms outlined above.

## 3 Health Benefits of RES

### 3.1 Degenerative Musculoskeletal Diseases

**FIGURE 2:**
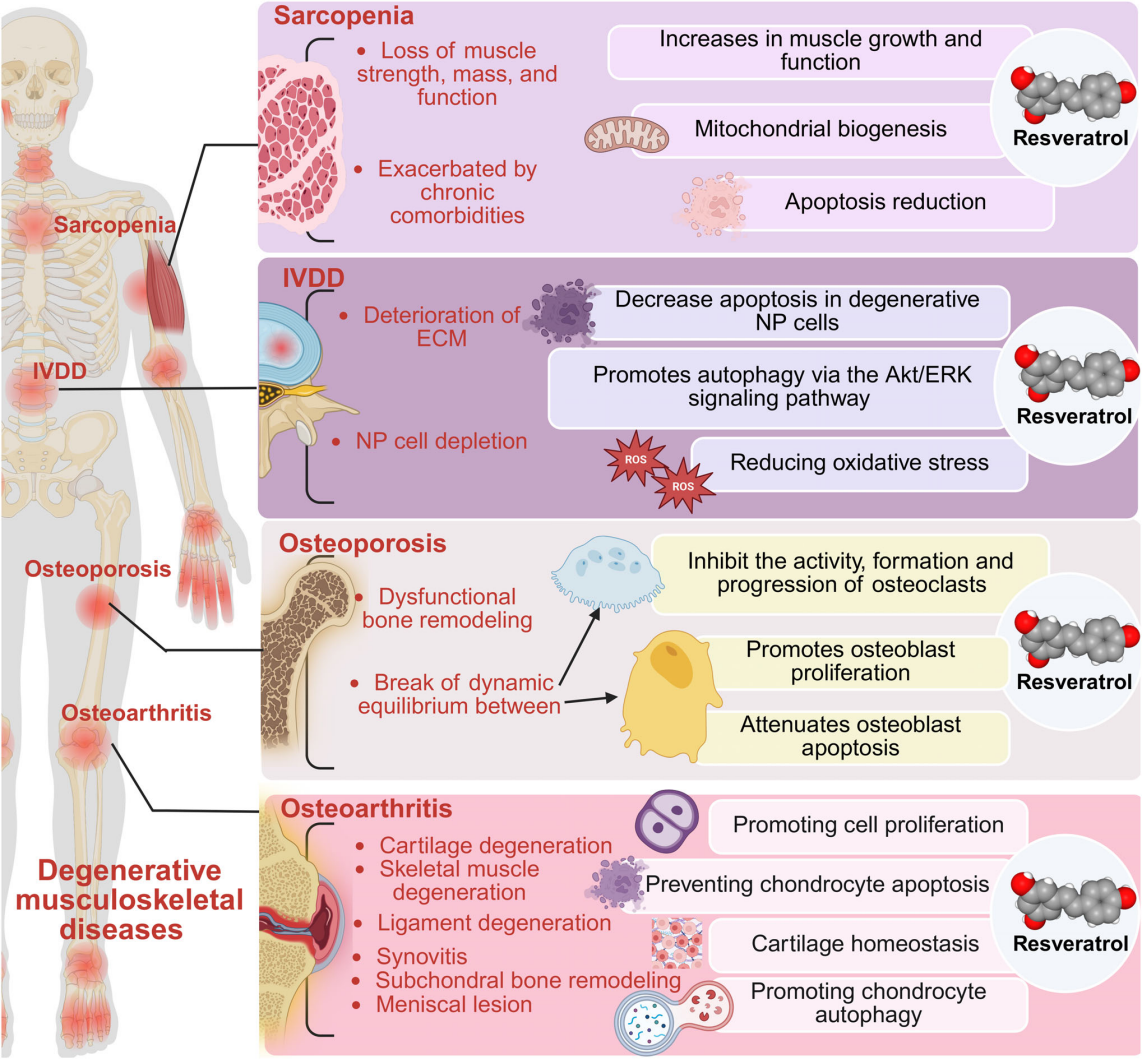
RES in degenerative musculoskeletal diseases. AKT, protein kinase B; ECM, extracellular matrix; ERK, extracellular signal‐regulated kinase; IVDD, intervertebral disc degeneration; NP cell, nucleus pulposus cell.

Degenerative musculoskeletal diseases encompass a range of chronic conditions associated with the aging and deterioration of the structural integrity and function of bones, cartilage, and muscles, including osteoarthritis (OP), osteoporosis (OS), intervertebral disc degeneration (IVDD), and sarcopenia (SP). As the global population ages, the impaired mobility, chronic pain, psychological burden, and both direct and indirect economic costs imposed by these diseases have a profound impact on life expectancy and quality of life, particularly in economically developing regions. Numerous recent studies have highlighted the diverse benefits of RES for degenerative musculoskeletal diseases, although debates persist regarding its bioavailability, optimal dosage, and potential adverse effects (Figure 2).

#### 3.1.1 Osteoarthritis (OA)

OA is a comprehensive joint disease characterized by cartilage degeneration, subchondral bone remodeling, synovitis, meniscal lesions, ligament degeneration, and skeletal muscle deterioration.

RES holds significant potential in improving OA by promoting chondrocyte proliferation, preventing chondrocyte apoptosis, and maintaining a dynamic balance between the anabolic and catabolic processes of cartilage. RES enhances chondrocyte proliferation by upregulating the HO‐1/Nrf2 [52] pathway and inhibiting the PI3K/AKT signaling pathway [53]. This process also appears to be facilitated by inhibiting TNF‐β‐induced proinflammatory responses and the NF‐κB signaling pathway [54]. Additionally, RES prevents chondrocyte apoptosis by inhibiting the synthesis of adenosine triphosphate (ATP) and prostaglandin E2 (PGE2) [55]. Animal studies have shown that RES inhibits the secretion of IL‐1β, TNF‐α, IL‐6, and NO and prevents apoptosis in articular chondrocytes [56]. Intra‐articular injection of RES can regulate the expression of HIF‐1α and HIF‐2α, which in turn modulates the AMPK/mTOR signaling pathway, delaying articular cartilage degeneration and promoting chondrocyte autophagy in destabilization of the medial meniscus (DMM)‐induced OA mice [57]. By blocking the JNK/ERK–AP‐1 pathway, RES inhibits the production of MMP‐13 induced by advanced glycation end products and prevents type II collagen degradation in a porcine cartilage explant model [58]. This process also seems to influence NF‐κB, thereby affecting the expression of MMPs, by acting directly on articular chondrocytes [59].

In synovitis associated with OA, RES may inhibit the proliferation of fibroblast‐like synoviocytes by activating the SIRT1/Nrf2 signaling pathway [60] or by inhibiting the MAPK and NF‐κB signaling pathways [61, 62, 63]. The osteoprotective effect of RES in subchondral bone remodeling is linked to the restoration of antiapoptotic signaling and the regulation of apoptosis, which is achieved through the upregulation of BCL‐2 and the downregulation of caspase‐3 [64]. It is important to note that research on the effects of RES on ligament degeneration and meniscus injury remains limited. Given RES's anti‐inflammatory and antioxidant properties, including its ability to scavenge reactive oxygen species (ROS), there is considerable potential for further investigation into its application in meniscus replacement materials and the treatment of ligament degeneration.

#### 3.1.2 Osteoporosis (OP)

Dysfunctional bone remodeling, which refers to the disrupted balance between osteoblast‐mediated bone formation and osteoclast‐mediated bone resorption, can lead to bone loss and is the primary cause of OP [65].

RES can inhibit the activity, formation, and progression of osteoclasts. While RANKL enhances the functions of p300 and NF‐κB signaling in osteoclasts, RES reverses the activation of the RANKL–p300–NF‐κB pathway, thereby reducing bone resorption [66]. Further research has shown that by blocking the PI3K/AKT signaling pathway, RES enhances the transcriptional activity of FOXO1, which in turn boosts tolerance to oxidative stress and inhibits osteoclastogenesis. Additionally, RANKL may suppress the PI3K/AKT signaling pathway [67].

RES also affects osteoblast differentiation and mineralization, thereby inhibiting OP. It enhances the interaction between SIRT1 and FOXO3a, reduces the acetylation of FOXO3a, and promotes its nuclear translocation. This process, in turn, stimulates the proliferation and osteogenic differentiation of bone marrow mesenchymal stem cells while inhibiting their senescence [68]. Furthermore, RES may enhance the Wnt/β‐catenin signaling pathway and regulate FOXO transcriptional activity through SIRT1. The Wnt signaling pathway not only promotes osteoblast proliferation but also mitigates osteoblast apoptosis [69, 70].

#### 3.1.3 Intervertebral Disc Degeneration （IVDD）

Intervertebral discs consist of three major components: the inner nucleus pulposus (NP), the outer annulus fibrosus (AF), and the cartilaginous endplates (CEP) [71]. It is widely recognized that the degradation of the extracellular matrix and the depletion of NP cells initiate and accelerate the progression of IVDD [72]. RES may reduce apoptosis in degenerative NP cells and increase the protein expression of Beclin‐1 and LC3‐II/I, promoting autophagy [73]. Furthermore, RES inhibits TNF‐α‐induced MMP‐3 expression in human NP cells by activating autophagy through the AMPK/SIRT1 signaling pathway [74]. It is important to emphasize that mildly deteriorated NP cells may serve as a critical target for molecular biological intervention in disc degeneration. SIRT1 promotes autophagy through the AKT/ERK signaling pathway, thereby protecting these mildly degraded human NP cells from apoptosis [75]. RES can also participate in the IVDD process by mitigating NP cell apoptosis. In a sodium nitroprusside‐induced apoptosis model of NP cells, RES protects against cell death by scavenging ROS but not NO [76]. This aligns with previous findings on the antioxidant mechanisms of RES and may also involve the activation of the PI3K/AKT pathway [77] and partial inhibition of the ERK1/2 pathway [78].

In the AF, RES reduces TNF‐α‐induced apoptosis in AF cells by decreasing ROS levels and enhancing SOD activity. In vitro, this effect underscores RES's ability to mitigate apoptosis through the reduction of oxidative stress [79]. In the CEP, studies have shown that RES therapy reduces apoptosis in CEP cells, suppresses TNF‐α production, and elevates IL‐10 levels, indicating its anti‐inflammatory and protective effects in IVDD [80]. In summary, research on RES's effects on the AF and CEP remains limited and primarily centers on its anti‐inflammatory properties, likely due to the more pronounced degeneration of the NP observed during IVDD.

#### 3.1.4 Sarcopenia (SP)

SP is characterized by the progressive loss of muscle strength, mass, and function, a condition often worsened by chronic comorbidities such as CVDs [81]. As a prominent activator of sirtuins, RES exhibits variable effects in intervention studies targeting SP. Sirtuins are increasingly recognized as promising therapeutic targets for SP. RES supplementation has been associated with enhanced muscle growth and function, along with cellular benefits such as increased mitochondrial biogenesis and reduced apoptosis—partially mediated through the PKA/LKB1/AMPK signaling pathway [82, 83]. RES can also combat sarcopenia by mitigating muscle inflammation, thereby contributing to the preservation of muscle mass and function [84]. However, contrary to these findings, some studies have reported that despite its antioxidative properties, RES fails to mitigate sarcopenia in aged mice [85], prevent the loss of plantar muscle mass in old rats subjected to 14 days of hindlimb suspension [86], or enhance the satellite cell response to mechanical overloading [87]. These discrepancies in findings and conclusions may stem from variations in the degree of aging in the experimental models and the criteria used to assess sarcopenia.

### 3.2 CVDs

**FIGURE 3:**
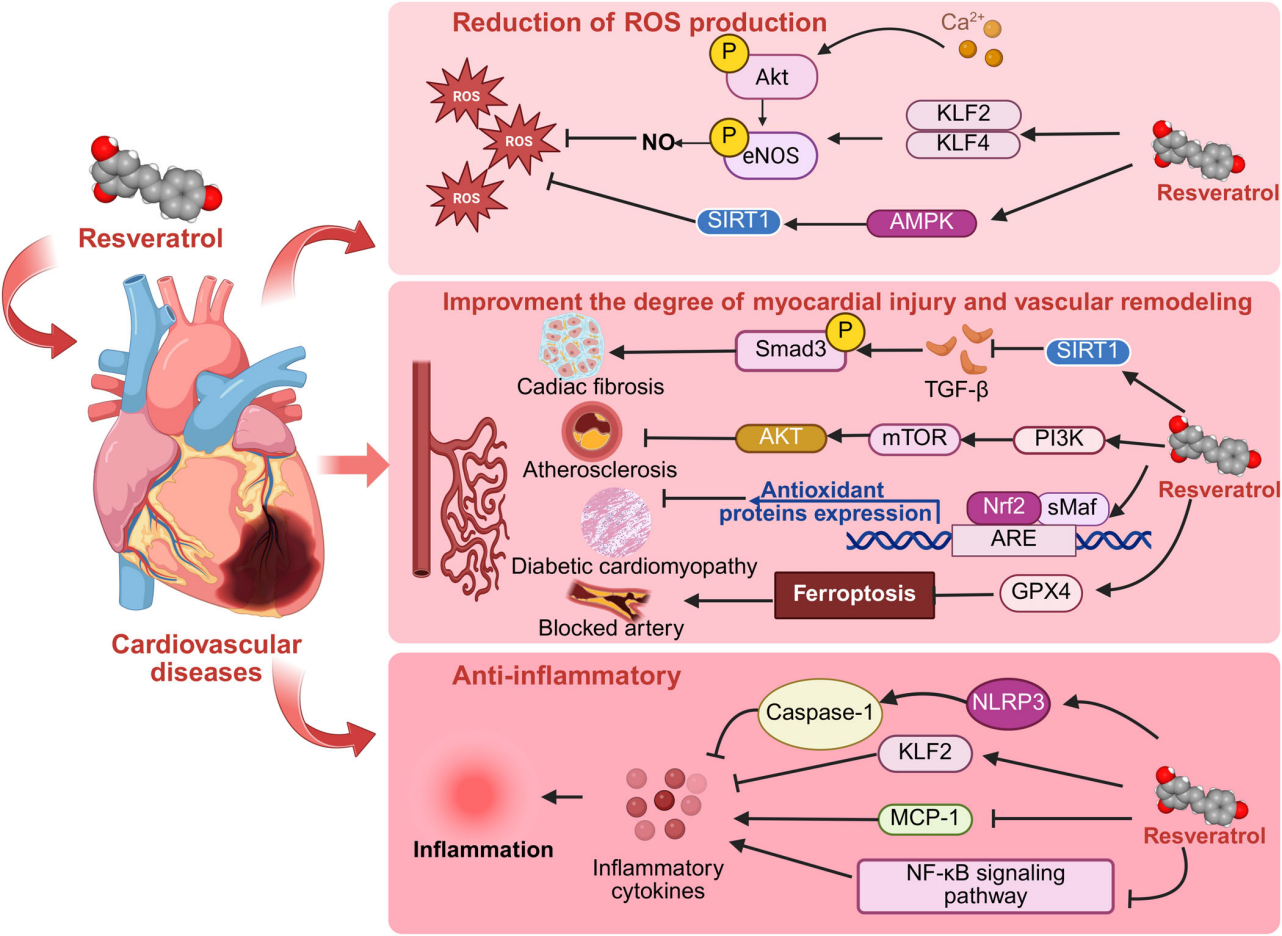
RES in CVDs. AKT, protein kinase B; AMPK, AMP‐activated protein kinase; ARE, antioxidant response element; eNOS, endothelial nitric oxide synthase; GPX4, glutathione peroxidase 4; KLF2, Krüppel‐like factor 2; MCP‐1, monocyte chemoattractant protein‐1; mTOR, mammalian target of rapamycin; NLRP3, NOD‐like receptor thermal protein domain associated protein 3; NF‐kB, nuclear factor‐kappa B; Nrf2, nuclear factor erythroid 2‐related factor 2; PI3K, phosphoinositide 3‐kinase; ROS, reactive oxygen species; SIRT1, sirtuin 1; sMaf, small Maf proteins.

CVDs encompass a range of noncommunicable diseases that impact the heart or blood vessels, including hypertension, heart failure, coronary artery disease, cerebrovascular disease, cardiomyopathies, and peripheral arterial disease [88]. The “French paradox” initially sparked scientific interest in RES, as it is abundantly present in red wine and may help alleviate arterial wall degradation caused by oxidative stress from chronic inflammation—such as atherosclerosis [89]. RES is also believed to play a role in the metabolic and molecular changes associated with endothelial and myocardial dysfunction [90] (Figure 3).

The cardiovascular protective effects of RES are primarily manifested through its ability to scavenge ROS or reduce ROS production, particularly during the process of endothelial dysfunction. Endothelial nitric oxide synthase (eNOS) is a Ca^2^⁺/calmodulin‐dependent enzyme in endothelial cells that catalyzes the conversion of arginine to citrulline, subsequently promoting the production of NO. NO can help mitigate atherosclerosis by regulating vascular tone and promoting improved blood flow [91]. RES can prevent excessive ROS generation by directly scavenging ROS through the AKT/eNOS pathway or indirectly by modulating NADPH oxidase 1 (NOX1) [92, 93]. RES also appears to inhibit the detrimental effects of ROS on cells by modulating mitochondrial metabolism and ATP production through the AMPK/SIRT1 pathway or by suppressing the increase in cellular NAD+ levels [94, 95]. Additionally, RES improves endothelial dysfunction by increasing NO levels in the endothelium. This process involves the upregulation of GTP cyclohydrolase 1, which enhances tetrahydrobiopterin biosynthesis to prevent eNOS uncoupling, as well as directly increasing eNOS phosphorylation and expression, ultimately stimulating NO production [96, 97, 98]. Simultaneously, RES acts as an activator of the transcription factors Krüppel‐like factor‐2 (KLF2) and KLF4, which regulate eNOS gene expression. RES enhances eNOS expression through the KLF2/4 pathway [99].

RES can also alleviate myocardial injury and vascular remodeling. Studies have shown that RES significantly inhibits the expression of ferroptosis‐related signals and mitigates mitochondrial damage by inducing SIRT1/GPX4 or KAT5/GPX4 pathways, ultimately improving outcomes in myocardial infarction [100]. By targeting abnormal proliferation following vascular injury, RES can inhibit smooth muscle cell proliferation through the PI3K/AKT/mTOR pathway, thereby improving conditions such as atherosclerosis and pulmonary hypertension [100, 101]. RES increases Nrf2 expression and transcriptional activity, along with the activation of downstream antioxidant targets of Nrf2, thereby helping to prevent myocardial injury induced by diabetes mellitus (DM) [102]. RES significantly alleviates cardiac oxidative injury induced by fenitrothion and repaired the transcript levels of SIRT1, c‐JNK, and caspase‐9/3, along with p53 immunoreactivity [103]. RES treatment significantly improves left ventricular function and reduces left ventricular hypertrophy and cardiac fibrosis in pressure overload rats by regulating the SIRT1/TGF‐β1/p‐Smad3 signaling pathway [104]. RES also protects the heart by downregulating glucose metabolism enzymes or increasing the expression of SIRT3, which facilitates the deacetylation of cyclophilin D in myocytes affected by pulmonary arterial hypertension, thereby regulating the openness of the mitochondrial permeability transition pore (mPTP) [105, 106]. RES can regulate heart failure‐induced expressions of Foxo1b and FOXO3a to normal levels. It significantly alleviates heart failure, including rescuing abnormalities in heart rate, blood flow, cardiac output, and NPPB overexpression [107].

RES antagonizes the occurrence of cardiovascular inflammation through various signaling pathways. It significantly inhibits TNF‐α‐induced late endothelial progenitor cell inflammatory damage by upregulating KLF2 expression and downregulating the expression of intercellular adhesion molecule 1 and monocyte chemoattractant protein‐1 [108]. RES can reduce the activation of NF‐κB‐induced inflammatory factors by inhibiting the mRNA and protein expression of the NLRP3 inflammasome and caspase‐1. It also downregulates a variety of inflammatory cytokines, thereby playing an anti‐inflammatory role in the cardiovascular complications associated with DM [109, 110].

### 3.3 Anticancer Properties

**FIGURE 4:**
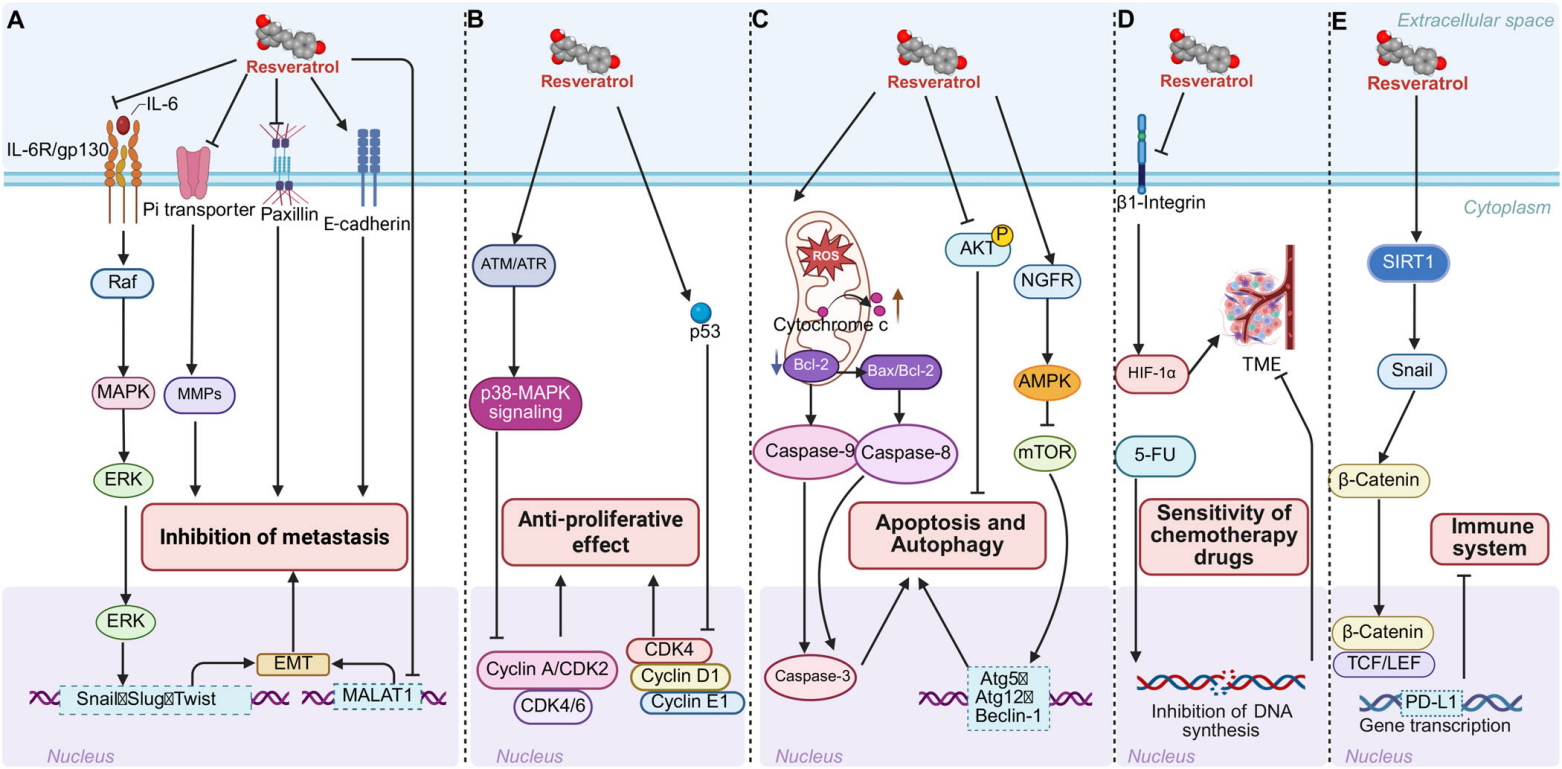
RES in cancer. RES has the potential to inhibit cancer progression by reducing cancer cell migration and proliferation, promoting apoptosis, and enhancing the sensitivity of chemotherapy drugs. 5‐FU, 5‐fluorouracil; AMPK, AMP‐activated protein kinase; ATR, ataxia telangiectasia and Rad3‐related protein; ATM, ataxia telangiectasia mutated; Bax, Bcl‐2‐associated X protein; Bcl‐2, B‐cell lymphoma 2; CDK4/6, cyclin‐dependent kinase 4/6; EMT, epithelial–mesenchymal transition; ERK, extracellular signal‐regulated kinase; GPX4, glutathione peroxidase 4; HIF‐1α, hypoxia‐inducible factor‐1α; IL‐6, interleukin‐6; KLF2, Krüppel‐like factor 2; LEF, lymphoid enhancer factor; MALAT1, metastasis‐associated lung adenocarcinoma transcript 1; MAPK, mitogen‐activated protein kinase; MCP‐1, monocyte chemoattractant protein‐1; mTOR, mammalian target of rapamycin; NGFR, nerve growth factor receptor; Nrf2, nuclear factor erythroid 2‐related factor 2; PD‐L1, programmed death‐ligand 1; PI3K, phosphoinositide 3‐kinase; p53, tumor protein p53; RAF, rapidly accelerated fibrosarcoma; SIRT1, sirtuin 1; Slug, snail family transcriptional repressor slug; Snail, snail family transcriptional repressor snail; TCF, T‐cell factor; TME, tumor microenvironment; Twist, Twist family BHLH transcription factor; Atg5, autophagy‐related 5; MMPs, matrix metalloproteinases.

Numerous studies have highlighted the multifaceted nature of RES in tumor inhibition, demonstrating its effects through various mechanisms rather than a singular pathway. RES has been shown to play a crucial role in the development of several cancers, including colon, lung, breast, prostate, liver, and pancreatic cancers, particularly those associated with obesity. RES is also regarded as a potent natural chemopreventive agent, complementing conventional chemotherapy and serving as a combination therapeutic with other treatments. The primary mechanisms by which RES combats cancer include halting the cell cycle, inhibiting cancer cell proliferation, reducing metastasis, promoting apoptosis and autophagy, modulating the immune system, and enhancing the efficacy of chemotherapy (Figure 4).

RES effectively induces cell cycle arrest and suppresses the proliferation of cancer cells. It achieves this by arresting the cell cycle at the S phase and triggering DNA‐damage‐induced apoptosis through the activation of p38–MAPK signaling [111] in oral and colorectal cancer cells [111, 112]. Interestingly, by upregulating the expression of phosphorylated histone H2AX (γ‐H2AX) and cleaved caspase‐3, RES downregulates p38/MAPK signaling, induces G1‐to‐S phase cell cycle arrest, and reduces cell survival in triple‐negative breast cancer cells (TNBCs) [113]. This suggests that the specific mechanism of RES in different cancer cells is not single. After treatment with RES, the breast cancer 4T1 cells undergo S phase arrest, as evidenced by an increased percentage of cells in the S phase and a corresponding decrease in the G1/G0 phase [114]. Moreover, the combination of RES and docetaxel induces cell cycle arrest in prostate cancer C4‐2B cells by stimulating the expression of p53 and suppressing the levels of CDK4, cyclin D1, and cyclin E1 [115]. RES analogues consistently reduced the pancreatic cancer cell subpopulation, exhibiting a CD133+EpCAM+ stem‐like phenotype, while simultaneously exerting dramatic effects on cell clonogenicity, with minimal toxicity observed in normal HFF‐1 cell [116]. RES inhibited cholangiocarcinoma cell proliferation, triggered apoptosis alongside autophagy, and significantly diminished the presence of cancer‐associated fibroblasts and the production of IL‐6 [117].

RES can reduce the metastasis of cancer cells. RES dose‐dependently inhibited the migration‐promoting adhesion adapter protein paxillin while simultaneously enhancing the expression of E‐cadherin, a key factor in the phenotypic transformation and invasion of colorectal cancer cells. β1‐Integrin likely plays a central role in this process [118]. RES holds the potential to prevent IL‐6‐induced gastric cancer metastasis by inhibiting the activation of the Raf/MAPK signaling pathway [119]. RES hindered migration and invasion in human gastric cancer cells by suppressing the MALAT1‐mediated EMT [120]. In breast cancer cells with a propensity for metastasis, RES noncompetitively inhibited the Na+‐dependent Pi transporter, thereby restraining the adhesion and migration of human breast cancer cells, effectively preventing their metastatic progression [121]. This process also includes upregulating the expression of E‐cadherin while downregulating the levels of matrix metalloproteinase (MMP)‐2, MMP‐9, and vimentin [122]. RES suppressed the proliferation and metastasis of pancreatic cancer cells by inhibiting the expression of ryanodine receptor type 2 and enhancing the expression of phosphatase and tensin homolog [123]. RES inhibits the hepatocyte growth factor‐mediated interaction between the stroma and epithelium while also suppressing epithelial prostate cancer cell migration by attenuating the regulation of EMT [124].

RES can promote apoptosis and autophagy of cancer cells. RES induces apoptosis in colorectal cancer cells via a ROS‐mediated mitochondrial apoptotic pathway, elevating ROS levels and the expression of cytochrome *c*, cleaved caspase‐9, and cleaved caspase‐3, while simultaneously reducing Bcl‐2 expression [125]. In RES‐treated breast cancer cells, the Bax/Bcl‐2 ratio decreases while caspase‐8 activity increases, thereby activating the extrinsic apoptotic pathway [126]. RES induces apoptosis in TNBCs by downregulating the mRNA expression of polymerase delta 1. RES disrupts lung cancer cellular homeostasis by depleting the intracellular antioxidant pool, thereby increasing ROS production. This leads to a concentration‐ and time‐dependent increase in the number of senescent and apoptotic cells [127]. Treatment with high‐concentration (>10 µM) RES downregulates SIRT1 expression, enhances p53 acetylation, and elevates the expression of p21, Bax, cytochrome *c*, and caspase‐3, ultimately inducing apoptosis in colorectal cancer cells [128]. RES has been shown to induce autophagy and apoptosis in non‐small‐cell lung cancer cells by activating the nerve growth factor receptor (NGFR)–AMPK–mTOR pathway. Mutual promotion is observed between apoptosis and lethal autophagy. Conversely, cytoprotective autophagy promoted apoptosis without being influenced by it [129]. RES enhances the expression of autophagy‐related genes and proteins, promoting the formation of autophagosomes in breast cancer cells. It induces autophagy by upregulating SIRT3 expression and phosphorylated AMPK [130]. Furthermore, RES induces autophagy and apoptosis in cisplatin‐resistant oral cancer cells by enhancing phosphorylation of AMPK, inhibiting the AKT signaling pathway, and upregulating the expression of autophagic mRNA genes, including Atg5, Atg12, Beclin‐1, and LC3‐II [131].

RES can regulate the immune system [132]. RES activates SIRT1 deacetylase, which deacetylates and stabilizes the transcription factor Snail. Snail subsequently represses Axin2 transcription, leading to disassembly of the destruction complex and enhanced β‐catenin/TCF binding to the PD‐L1 promoter. Ultimately, RES inhibits PD‐L1 expression in lung cancer cells via the Wnt signaling pathway, thereby suppressing the T‐cell‐mediated immune response [133]. In the prevention of inflammation‐driven colorectal cancer, RES suppresses the proinflammatory T‐cell response by reducing Th1 and Th17 cell populations while enhancing the presence of anti‐inflammatory CD4⁺ FOXP3⁺ Tregs and CD4⁺ IL‐10⁺ cells [134]. Chimeric antigen receptor (CAR)‐engineered T cell therapies have emerged as potent and transformative approaches in cancer immunotherapy. However, the clinical application of CAR‐T therapy remains limited due to severe adverse effects in patients, primarily stemming from excessive cytotoxic activity and inadequate regulation of T cell responses. The RES–repressible CAR expression system enables effective suppression of T cell activation upon RES administration in both primary T cells and xenograft tumor mouse models, thereby enhancing patient safety [135].

RES can improve the sensitivity of chemotherapy drugs and offer potential radioprotection and radiosensitization (Table 1). Radiation therapy is widely employed in cancer treatment; however, its effectiveness is often hindered by radioresistance and adverse side effects. Consequently, the investigation of agents that can potentiate the therapeutic effects of radiation while safeguarding normal cells is of considerable significance [136]. RES sensitizes breast cancer cells to the PARP inhibitor talazoparib by concurrently inhibiting AKT signaling and autophagic flux. This dual inhibition compromises homologous recombination‐mediated repair of double‐strand breaks [137]. RES modulates chemosensitization to 5‐fluorouracil (5‐FU) through the β1‐integrin/HIF‐1α axis within the colorectal cancer tumor microenvironment (TME). This process diminishes TME‐driven vitality, proliferation, colony formation, invasive potential, and the mesenchymal phenotype, including promigratory pseudopodia [138]. RES holds promise as a radiosensitizer for breast cancer cells. The combination of 10 µM RES and 3 Gy ionizing radiation induced apoptosis in MCF‐7 breast cancer cells by reducing the Bax/Bcl‐2 ratio [126]. RES heightens the sensitivity of colorectal cancer cells to cetuximab by upregulating the expression and phosphorylation of connexin 43, thereby enhancing gap junction function. This process was implicated in the inhibition of the AKT pathway [139].

**TABLE 1:** Studies of RES in combination with other substances or treatment modalities for cancer treatment.

Combination	Type of cancer	Effect	Author	Year	References
RES and quercetin	Oral cancer	Cell growth inhibition, DNA damage, and S‐phase cell cycle arrest	Singh	2020	[112]
RES and capsaicin	Colorectal cancer	The radio‐sensitization of subcutaneous colorectal tumors with similar efficiency to 5‐FU and lower hematological toxicity	Samuel Amintas	2025	[140]
RES and cetuximab	Colorectal cancer	RES may sensitize colorectal cancer cells to cetuximab via upregulating connexin 43 to inhibit the AKT pathway.	Yijia Wang	2020	[139]
RES and peanut skin procyanidins	Colorectal cancer	The combination might exert synergistic anticancer effects by regulating AKT, ERK, and NF‐κB signaling pathways.	Na Wang	2023	[141]
RES, pharmacologic ascorbate and chloroquine	Pancreatic ductal adenocarcinoma	Synergistic cytotoxic effect	Kinga Makk‐Merczel	2024	[142]
RES and sirolimus	Lymphangioleiomyomatosis	The addition of RES was safe and well tolerated in patients	Nishant Gupta	2023	[143]
RES and tivozanib	Renal cell carcinoma	RES can prevent the proliferation of cancer cells and reduce the side effects of tivozanib	Diana Taheri	2024	[144]
RES and rapamycin	Breast cancer	The combination internalized in an estrogen receptor‐positive human breast cancer cell line and improved cytotoxicity.	Leidiana Rocha Dos Reis	2023	[145]
RES and tamoxifen	Breast cancer	The combination increased the expression of tumor inhibitor miRNA, which made cancer cells more sensitive to tamoxifen.	Aliaa M Radwan	2024	[146]
RES and Olaparib	Breast cancer	The combination inhibited PARP1 activity in the chromatin, resulting in deregulation of recombination pathway in breast cancer cells.	Saptarshi Sinha	2024	[147]
RES and pterostilbene	Prostate cancer	Stable, no systemic toxicity, high biodistributed/accumulated in prostate cells	Alok Nath Sharma	2024	[148]

### 3.4 Neuroprotective Effects

Neurological diseases (NDs), encompassing both neurodegenerative disorders and acute injuries, represent a broad spectrum of conditions affecting the brain, spinal cord, and peripheral nerves, often resulting in varied symptoms and significant functional impairments [149]. SIRT1 is widely expressed in brain regions commonly implicated in the pathology of NDs [150].

**FIGURE 5:**
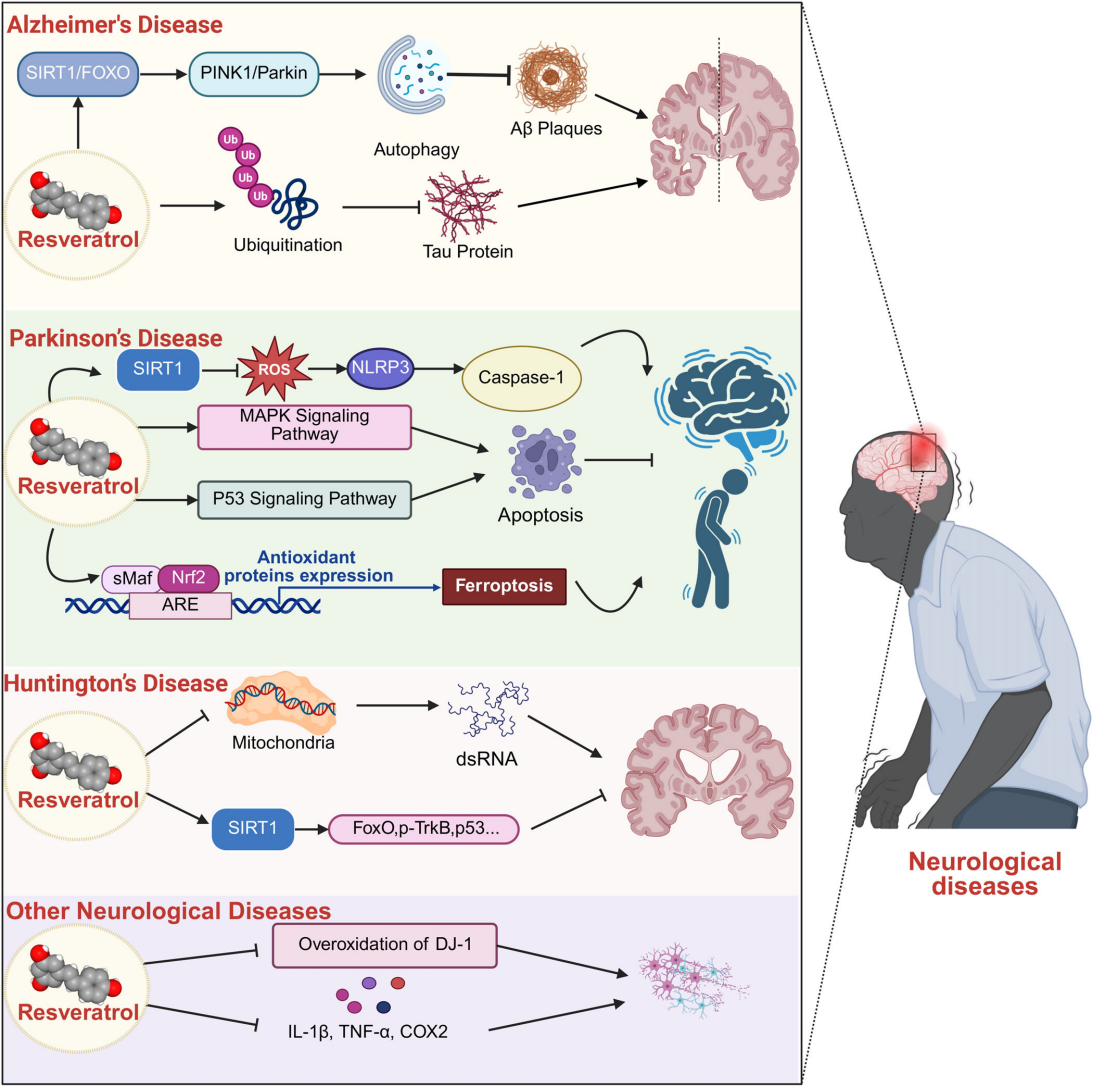
Neuroprotective effects of RES. ARE, antioxidant response element; COX2, cyclooxygenase‐2; FOXO, Forkhead box O; IL‐1β, interleukin‐1β; MAPK, mitogen‐activated protein kinase; NLRP3, NOD‐like receptor thermal protein domain associated protein 3; Nrf2, nuclear factor erythroid 2‐related factor 2; PINK1, PTEN‐induced putative kinase 1; ROS, reactive oxygen species; SIRT1, sirtuin 1; sMaf, small Maf; TNF‐α, tumor necrosis factor‐α; TrkB, tropomyosin receptor kinase B; dsRNA, double‐stranded RNA; p53, tumor protein p53.

RES, a potent SIRT1 activator, possesses the capacity to cross the blood–brain barrier (BBB), thereby conferring neuroprotective effects within the central nervous system [151] (Figure 5).

Neurodegenerative diseases—including Alzheimer's disease (AD), Parkinson's disease (PD), and Huntington's disease (HD)—are marked by aberrant protein aggregation, ultimately resulting in progressive neuronal damage [152]. For instance, the defining pathological features of AD include the accumulation of amyloid‐β (Aβ) aggregates forming senile plaques and the aberrant hyperphosphorylation of Tau protein, which leads to the formation of neurofibrillary tangles within neuronal cells [153]. Additionally, mitochondrial dysfunction—closely linked to the onset of neurodegenerative diseases—can diminish glucose and oxygen metabolism in the brain and disrupt the function of the respiratory chain [152]. In AD, RES plays a pivotal role in preventing Aβ aggregation by hindering the formation of larger oligomeric assemblies from smaller molecular aggregates and disrupting preexisting Aβ plaques [154]. By activating SIRT1, RES directly reduces levels of Aβ peptides and amyloid precursor protein‐derived C‐terminal fragments through autophagy in neurons [155]. Additionally, RES stimulates the SIRT1–FOXO axis, thereby decreasing Aβ plaque accumulation and reversing mitochondrial dysfunction [156]. RES can also ameliorate AD by attenuating inflammation in affected regions through multiple pathways, including the glycogen synthase kinase‐3β signaling pathway and the NF‐κB signaling cascade [157, 158]. Inhibition of SIRT1 can exacerbate tau accumulation by increasing its acetylation and reducing its ubiquitination in primary neurons and transgenic HEK293T cells [159]. In PD, RES regulates iron metabolism and mitigates ferroptosis in PD models via the SIRT1/Nrf2 signaling pathway [160]. Additionally, the activation of the NLRP3 inflammasome induced by subarachnoid hemorrhage is inhibited by SIRT1 [161]. RES can enhance the autophagic degradation of α‐synuclein and elevate the level of LC3 II, a key marker of autophagy [162]. RES may contribute to the induction of apoptosis by modulating the p53 and MAPK signaling pathways [163, 164]. HD is a hereditary, autosomal‐dominant neurodegenerative disorder caused by a mutation in the huntingtin protein, leading to toxic aggregations in the brain [165]. RES may treat HD by reversing the downstream response to immunogenic stressors that elevate mitochondrial RNA expression. These mitochondrial double‐stranded RNAs are closely linked to the pathogenesis of HD [166]. Moreover, SIRT1 can regulate various physiological and pathological processes by modulating the activity of multiple targets, including FOXO3a, phospho‐tropomyosin receptor kinase B, and p53, thereby mediating neuroprotection in HD models [167]. Collectively, these findings underscore the potential of RES in the treatment of neurodegenerative diseases.

In the other NDs other than neurodegenerative diseases, RES also plays an important role in rehabilitation potential. Pre‐administration of RES to I/R rats significantly reduces the infarction area, oxidative stress, inflammation, and apoptosis. The overoxidation of DJ‐1 protein is a critical factor contributing to post‐I/R cerebral damage. RES mitigates the reduced levels of oxidized DJ‐1, promoting its reduction and activating the PI3K/AKT survival pathway [168]. RES effectively inhibits the upregulation of inflammatory factors, including IL‐1β, TNF‐α, and COX2 mRNA expression. Administration of RES significantly reduces neurological deficiency scores, cerebral water content, and the enzymatic activity of myeloperoxidase [149, 169]. Additionally, RES demonstrates effectiveness in reducing seizures and increasing latency in epilepsy, lowering brain malondialdehyde levels, and preventing kainic acid‐induced seizures. These findings suggest that RES can serve as an adjuvant for antiepileptic treatment, likely due to its potent ability to scavenge ROS [170]. RES decreases brain infarct volume, neuronal damage, and neuronal death. However, these protective effects are diminished when the PI3K/AKT and JAK2/STAT3 pathways are inhibited [149]. In vitro, RES suppresses M1 microglia polarization while promoting M2 microglia polarization. It alleviates brain damage by reducing BBB permeability in PRV‐infected mice and decreasing the expression of MMP‐2, MMP‐9, and Zonula Occludens Protein‐1 in the cortex [171]. RES reduces both basal and LPS‐stimulated MMP levels, as well as cerebrospinal fluid levels of tissue inhibitor of metalloproteinases‐1, released from cultured microglia and astrocytes. However, neuroplasticity‐promoting MMP release from neurons remains unaffected. This highlights the diverse actions of RES, which vary according to cell types and molecular targets [172]. Although trans‐RES has garnered more scientific attention, cis‐RES also exhibits protective effects against neuronal DNA oxidative damage, seemingly producing the opposite effect of trans‐RES. Cis‐RES binds to tyrosyl‐tRNA synthetase (TyrRS), mimicking a “tyrosine‐free” conformation, which enhances TyrRS activity, facilitates histone serine‐ADP‐ribosylation‐dependent DNA repair, and provides neuroprotection in a TyrRS‐dependent manner. In contrast, trans‐RES inhibits serine‐ADP‐ribosylation‐dependent DNA repair, leading to neurodegeneration in rat cortical neurons [173]. This suggests that RES, in its different conformations, may yield divergent outcomes in the context of nervous system diseases. While most current studies have not yet investigated the varying effects of the two forms in different diseases, the potential for opposite results underscores the importance of addressing this distinction in future research.

### 3.5 Antiaging Effects

The aging of the global population has heightened interest in understanding the aging process and developing strategies to extend a healthy lifespan. Aging is characterized by alterations in epigenetic modifications and, much like all living systems, is accompanied by entropy. Aging is characterized by several markers, including senescence‐associated β‐galactosidase activity, telomere‐related DNA damage, and the expression of cell cycle inhibitors such as p16 and p21 [174]. However, it is important to note that aging is not entirely detrimental to the body. Studies have shown that the elimination of senescent cells not only fails to improve health conditions but can also lead to the emergence of other adverse symptoms. For example, the continuous or acute removal of senescent vascular endothelial cells in mice disrupted blood–tissue barriers, leading to the accumulation of blood‐borne macromolecular waste. This resulted in perivascular fibrosis across various tissues, ultimately contributing to health deterioration [175]. Senescence is likely pleiotropic, meaning its effects can be both beneficial and harmful in humans, depending on the context [174]. But no doubt, delaying aging appeals to countless researchers. RES, one of the most significant biologically active phenolic compounds, has been demonstrated to mitigate aging through various mechanisms. Combining various basic and clinical trials, RES has been found to be an effective and beneficial treatment for aging and aging‐related disorders (AD [173, 176, 177], OP [178, 179, 180], type 2 diabetes [181, 182, 183], etc.).

RES can enhance telomerase activity to counteract aging. In age‐associated infertility, RES‐treated mice exhibit a larger follicle pool, increased telomerase activity, and longer telomeres [184]. This process may be facilitated by the positive regulation of telomere length through the activation of SIRT1 [185, 186]. The short telomere zebrafish line (ST2) larvae exhibit reduced telomerase expression and activity, accompanied by shortened telomeres. Studies confirm the antiaging properties of RES in ST2, which enhance telomere maintenance [187].

RES can ameliorate mitochondrial dysfunction and protect mitochondria in senescent cells. Targeting senescence‐associated mitochondrial dysfunction has been proposed as a potential mechanism for senolytic drugs. RES enhances mitochondrial biogenesis by modulating its key effectors through various mechanisms, including PGC‐1α, SIRT1, estrogen‐related receptor‐α, and telomerase reverse transcriptase [188]. PGC‐1α is a key coordinator of mitochondrial biogenesis and serves as a target of SIRT1 [94]. RES appears to enhance mitochondrial activity by activating the AMPK–SIRT1–PGC1α axis, thereby upregulating the expression of genes associated with oxidative phosphorylation [189, 190]. As demonstrated in animal studies, RES slows aging and extends life expectancy by activating sirtuins and AMPK, key regulators of energy metabolism and the aging process [191, 192]. By elevating cellular NAD+ levels and promoting energy catabolism, AMPK stimulates autophagy and mitochondrial biogenesis [193]. Senescent cells exhibit increased susceptibility to RES‐induced mitochondrial Ca2+ overload and subsequent cell death [194]. RES preserves mitochondrial integrity by inhibiting mitophagy and preventing mPTP opening via the AMPK–Mitofusin 2 axis in myocardial cells [195]. Studies indicate that RES can enhance mitochondrial elongation and modulate mitophagy through the classical PINK1/Parkin‐mediated mitophagy pathway [196]. RES regulates mitochondrial homeostasis by activating SIRT1 and inhibiting the AKT/mTOR pathway, leading to a reduction in mitochondrial ROS levels while concurrently enhancing mitochondrial function [197]. Mitochondrial dysfunction and bioenergetic failure contribute significantly to the development of degenerative diseases. RES plays a crucial role in maintaining mitochondrial health, largely due to its antioxidant and anti‐inflammatory properties.

Epigenetics has been identified as a crucial factor in the development of aging and age‐related diseases. Hypermethylation or hypomethylation of DNA is commonly observed in various degenerative diseases. For instance, hypermethylation is prevalent in the midbrain of AD patients. RES can modulate epigenetic regulators, influencing histone methylation and acetylation, particularly at the BRCA1, p53, and p21CIP1 promoters in human breast cancer cell lines. Chromatin immunoprecipitation revealed that exposure to 20 µM RES significantly reduced the enrichment of repressive histone marks (H4R3me2s and H3K27me3) while increasing the abundance of activating histone marks (H3K9/27ac) within the proximal promoter region of BRCA1, p53, and p21 [198]. Supplementation of maternal mice with RES increased the methylation levels of the Nrf2 and NF‐κB gene promoters in offspring, leading to altered expression of target genes and elevated hydrogen peroxide levels. This maternal RES supplementation may help prevent cognitive decline in the offspring of senescent mice [199].

Due to its wide‐ranging effects, including anti‐inflammatory and antioxidative properties, RES plays a pivotal role in various aspects of the aging process, such as nutrition and energy metabolism [200, 201]. This complexity renders the antiaging mechanism of RES highly intricate.

## 4 Potential Adverse Effects of RES

### 4.1 Toxicity and Dosage

The cytotoxicity of RES is primarily influenced by its concentration. While RES generally acts as an antioxidant, excessive concentrations can lead to increased lipid peroxidation or DNA damage, thus inducing cytotoxicity. However, concentration is not the sole determinant; factors such as the form of RES entering the body and the specific type of cell involved may also play significant roles.

The primary mechanism of RES‐induced cytotoxicity involves the induction of DNA damage and intracellular oxidative stress. Exposure to RES (100 µM) for 6 h leads to significant formation of γ‐H2AX foci, a marker of DNA damage, as well as the occurrence of chromosome aberrations (CAs) in cells. The initial peak of CAs induced by RES may be linked to oxidative DNA breaks occurring during the G2‐M phase of the cell cycle [202]. RES is responsible for the selective inhibition of various mammalian DNA polymerases and acts as a potent inhibitor of ribonucleotide reductase [203, 204]. RES also appears to induce S‐phase arrest and cellular senescence, where DNA double‐strand breaks are significantly increased. Additionally, it activates the chemokine receptor C‐X‐C motif chemokine receptor 2 (CXCR2)–p53 axis [205]. In conclusion, RES may induce replication blocking caused by unpaired base damage, single‐strand breaks in DNA, or by inhibiting DNA replication‐related enzymes [202]. Although RES is typically known for its antioxidant properties, it can also induce intracellular oxidative stress. At higher concentrations, RES appears to increase the G0/G1 population by generating ROS, which is accompanied by an elevation in caspase‐3/7 activity, indicating the initiation of apoptotic pathways [142]. Tissue‐attainable doses of RES can increase the intracellular oxidative state, leading to mitochondrial membrane depolarization and ultimately inducing endothelial cell death. This suggests that while RES has beneficial effects at certain concentrations, excessive doses may cause cellular stress and damage, particularly in endothelial cells [206]. Currently, the oxidant properties of RES are being utilized. For instance, when RES is combined with copper, it can deactivate cell‐free chromatin particle by generating ROS. These ROS, released from billions of dying cells daily, subsequently enter the bloodstream, where they wreak havoc on healthy cells [207].

Biphasic concentration‐dependent effects appear to be a critical factor contributing to the cytotoxicity of RES. At low concentrations, such as 0.1 µM, RES promotes embryonic development, whereas higher concentrations (>1 µM) induce detrimental effects, including cleavage arrest and embryonic death [208]. A study reported that a high dose of RES (1 g/day) significantly elevated certain biomarkers associated with the development of CVDs in overweight older adults (65 years and above) [209]. At high doses (1 g/day or higher), RES demonstrated systemic inhibition of cytochrome P450 (CYP), particularly hepatic CYP enzymes. Notably, despite RES's inhibitory effect on CYP, no in vivo metabolites of RES (such as RES‐3‐sulfate) have been found to exhibit similar effects [210].

While the potential toxicity of RES at high doses is a valid concern, it is important to acknowledge its rapid metabolism. The actual plasma and tissue concentrations, as well as the duration and dosage of RES in in vitro experiments, need significant considerations. Without specialized carriers or administration methods, RES may undergo swift metabolism in the intestine and liver. Consequently, the toxicity of the metabolites produced requires careful evaluation when assessing the dose and toxicity of RES. Furthermore, the cytotoxic effects exhibited by RES hold considerable anticancer potential. Many of the mechanisms through which RES exerts its anticancer properties mirror the cytotoxic mechanisms it employs, such as DNA damage induced by RES in combination with copper, which may contribute to its cytotoxic action against cancer cells [211].

### 4.2 Interaction with Medications

As a therapeutic agent, the intervention effects of RES inevitably influence the efficacy of other medications. For instance, RES serves as an effective chemical sensitizer for colorectal cancer cells, enhancing their responsiveness to chemotherapeutic drugs such as irinotecan and 5‐FU, among others [212]. RES, as a potential inhibitor of CYP enzymes, has been shown to elevate the plasma concentration–time curve and maximum concentration of various drugs, including cisapride, cyclosporine, felodipine, and midazolam. This effect is particularly pronounced when RES is consumed alongside grapefruit juice, which contains additional CYP inhibitors, further enhancing the drug's bioavailability [210]. Furthermore, RES acts as an inhibitor of intestinal CYP3A4; however, there is a paucity of studies to conclusively determine whether co‐administration of RES with CYP3A4 substrates could lead to adverse effects [213]. The capacity of RES to decrease the systemic clearance of oral nicardipine while exerting minimal impact on the pharmacokinetic parameters of intravenous nicardipine may be attributed to its inhibition of CYP3A4. Although this enhances the bioavailability of nicardipine, caution should be exercised in adjusting its dosage to mitigate potential toxic side effects [214]. Studies have demonstrated that RES can interact with drugs metabolized by UGT1A1, CYP1A2, CYP2C19, CYP2E1, and CYP3A [215], including bedaquiline (an inhibitor of mycobacterial ATP synthase) and Erlotinib (a selective epidermal growth factor receptor inhibitor) [216, 217]. RES has been shown to enhance the intestinal absorption of methotrexate and reduce its renal clearance following intravenous administration in rats. This effect may be attributed to the inhibition of multidrug resistance‐associated protein 2, organic anion transporter 1 (OAT1), and OAT3 [218].

RES holds significant potential as a clinical therapeutic agent. It is crucial to investigate its potential interactions with other drugs to enhance the reliability of RES and provide valuable insights for the development of RES‐based pharmaceuticals. Currently, no reports indicate severe adverse reactions or toxicity when RES is combined with other drugs. However, most drug–drug interactions focus on the enhancement of RES's effects on other medications, which may alter the safe dosage of certain drugs and increase the risk of toxic side effects.

### 4.3 Hormonal Effects

RES can mimic hormones within the body and influence hormonal activity and related metabolic processes in various ways. Structurally, RES resembles both natural and synthetic estrogens, particularly due to the phenolic A ring characteristic of natural estrogens, and is therefore classified as a phytoestrogen. RES can directly bind to the nuclear estrogen receptor (ER), modulating its genomic activity. While the affinity of RES for ER isoforms is comparable, or even slightly superior, to that of some synthetic estrogens, it remains much weaker than that of natural estrogens. To effectively activate the receptor, micromolar concentrations of RES are required [219]. RES can directly interact with the ER, functioning as a mixed agonist/antagonist [219]. Animal growth, body weight, serum cholesterol levels, uterine growth, and the differentiation index are all unaffected by RES. However, at the highest dose, RES causes a modest increase in uterine weight and antagonizes the E2‐induced reduction in plasma cholesterol without impacting other physiological effects of E2 [220]. RES can inhibit key enzymes in the steroidogenic pathway, thereby interfering with steroidogenesis. However, it may enhance the level of active estrogen by modulating estrogen metabolism. When gavaged to rats at doses of 50 and 100 mg/kg for 2 days, RES reduces serum testosterone levels to approximately half of those observed in the placebo group [221]. RES inhibits corticosteroid 11β‐hydroxysteroid dehydrogenase type 1 in microsomal preparations from rodent adipose tissue and mouse 3T3‐L1 cells. This enzyme activates glucocorticoids in rodents by converting 11‐dehydrocorticosterone to corticosterone, and in humans, by transforming cortisone into cortisol. In mouse Leydig cells, RES reverses cAMP‐mediated progesterone synthesis in a dose‐dependent manner, with effective concentrations ranging from 10 to 50 µM. These effects are linked to decreased cAMP‐mediated promoter activity and reduced expression of the steroidogenic acute regulatory protein gene [222]. RES administration to mothers via oral gavage at a dosage of 10 mg/kg/day during lactation restores E2 levels in the offspring. This is achieved through the inhibition of various isozymes responsible for the hydroxylation, glucuronidation, and sulfation of E2. These findings suggest that RES counters the accelerated estrogen metabolism induced by chromium exposure and effectively restores ovarian E2 levels [223]. The inhibition of the enzyme and the subsequent reduction in the production of active corticosteroids may explain the beneficial effects of RES on central adiposity [224]. Treatment with RES appears to reduce ER expression. A study demonstrated that mice administered a low daily dose of 4 mg of RES in tap water for 15 weeks exhibited decreased ER expression in mammary tissue, an effect resembling resistance to endocrine therapy [225]. Moreover, RES antagonizes the estrogen (E2)‐mediated reduction of plasma cholesterol while leaving other physiological effects of E2 unaffected [220]. Postmenopausal OP results from estrogen deprivation, which accelerates osteoclast development and activation, leading to an imbalance in bone resorption and formation, ultimately causing rapid bone loss, particularly in the years preceding and following menopause [226]. RES exhibits an affinity for both ERα and ERβ, thereby acting as an estrogen agonist to stimulate osteoblastogenesis [70].

The discussion surrounding the hormonal effects of RES revolves around the concept of hormesis, where low doses typically offer protective benefits while high doses may have detrimental effects that worsen disease progression and morbidity. Currently, both low and high concentrations of RES demonstrate advantageous outcomes in cancer chemoprevention and treatment, respectively, through its cytotoxic properties. Undoubtedly, the bioavailability of RES is crucial in understanding its hormonal effects, encompassing factors such as uptake, absorption, distribution, and metabolism, as well as the variations between plasma and tissue levels of RES. While hormone‐like effects of natural compounds are not uncommon, both the quantity of intake and potential side effects must be carefully considered. Moreover, many studies continue to regard RES as a dietary supplement, implying its prolonged consumption at specific doses may not cause immediate harm. However, long‐term use could interfere with estrogen‐related metabolism and signaling pathways. Therefore, future preclinical and clinical trials are essential to assess the long‐term impact of RES on steroid hormone homeostasis, with a particular focus on the effects of higher RES concentrations, considering its bioavailability.

### 4.4 Gastrointestinal Disturbances

RES, as a significant dietary polyphenol, can modulate the composition of the intestinal microbiota, facilitate its bioconversion into active metabolites by intestinal bacteria, and influence the integrity of the intestinal barrier [227]. For example, RES administration enhanced the diversity and structure of the gut microbiota by promoting the abundance of beneficial probiotics and upregulating the expression of tight junction proteins. Additionally, RES significantly mitigated MeHg‐induced delays in neurobehavioral reflexes and reduced total mercury levels [228]. Nevertheless, adverse gastrointestinal effects from RES have still been reported.

The study revealed that doses of RES exceeding 2.5 g per day may lead to vomiting, diarrhea, and mild liver dysfunction in clinical trials [229]. In overweight and obese postmenopausal women, a daily 1 g dose of RES resulted in diarrhea in 30% of the subjects and an increase in total cholesterol in 27.5% of the participants [230]. In another human study, a dosage of 2 g of RES twice daily was well tolerated by healthy subjects; however, diarrhea was frequently observed in six out of eight participants [231]. However, in another study, patients with nonalcoholic fatty liver disease treated with a daily dose of 3 g of RES did not experience any gastrointestinal disturbances [232]. A combination of RES and copper reduced transplant‐related toxicities in patients with multiple myeloma undergoing high‐dose melphalan treatment. The use of RES did not exacerbate adverse reactions such as nausea and vomiting, and it decreased the incidence of oral ulcers in these patients [233].

It is worth noting that, despite variations in dose, administration method, potential adverse effects on the gastrointestinal tract, and interactions with other drugs, RES treatment did not result in significant adverse effects overall. The significant therapeutic potential of RES in degenerative diseases, inflammatory conditions, and various chronic ailments cannot be overlooked. However, it is undeniable that further well‐designed, large‐scale randomized controlled trials are essential to establish the optimal dose, duration, safety, drug interactions, and both short‐ and long‐term effects of RES, particularly in individuals of all ages, with special emphasis on the elderly.

## 5 Bioavailability and Clinical Studies

The bioavailability of RES has consistently been a major challenge preventing its widespread use as a clinical drug. RES is a lipophilic compound with limited water solubility (approximately 0.02–0.03 mg/mL [234]), which contributes to its range of bioavailability issues. Following oral administration, RES is efficiently absorbed in the jejunum and ileum. One study observed a 70% absorption rate for a 25 mg oral dose of RES [235]. Due to its metabolic characteristics, pharmacokinetic studies of trans‐RES have shown very low serum levels of unmetabolized RES following oral administration [236]. Regardless of the dose, the plasma half‐life of RES in humans typically ranges from 4 to 10 h [235, 237]. However, RES undergoes rapid and extensive metabolism in the gut and liver through passive diffusion or interaction with membrane transporters, leading to its low bioavailability. It appears that neither repeated administration nor dose escalation can significantly enhance the bioavailability of RES [229]. Metabolic processes in the gut and liver primarily involve glucuronidation, sulfation, and hydrogenation by gut bacteria [238]. The biological actions of the metabolites, their conversion back to the parent compound within cells or organs, and recirculation pathways, such as the enterohepatic circulation, each contribute to varying degrees and have been proposed as potential mechanisms underlying these effects [239]. The most prevalent RES metabolite in plasma following administration was RES‐3‐O‐sulfate, along with its sulfate‐glucuronide conjugate, both of which remained detectable for over 10 h postadministration [240].

Although RES can accumulate in specific tissues or organs at relatively high concentrations in rodents, comparable to those used in many in vitro experiments, the in vitro effective dose range of RES (micromolar range in cell culture medium) and its in vivo bioavailability (nanomolar range in blood) in humans appear to differ. This discrepancy complicates the determination of the actual biologically effective concentration range for human supplementation. As lipophilic molecules, RES levels in tissues persist longer than in plasma, offering a more accurate reflection of RES bioavailability. Consequently, further structural modifications to RES and the development of sustained‐release dosage forms are necessary.

**FIGURE 6:**
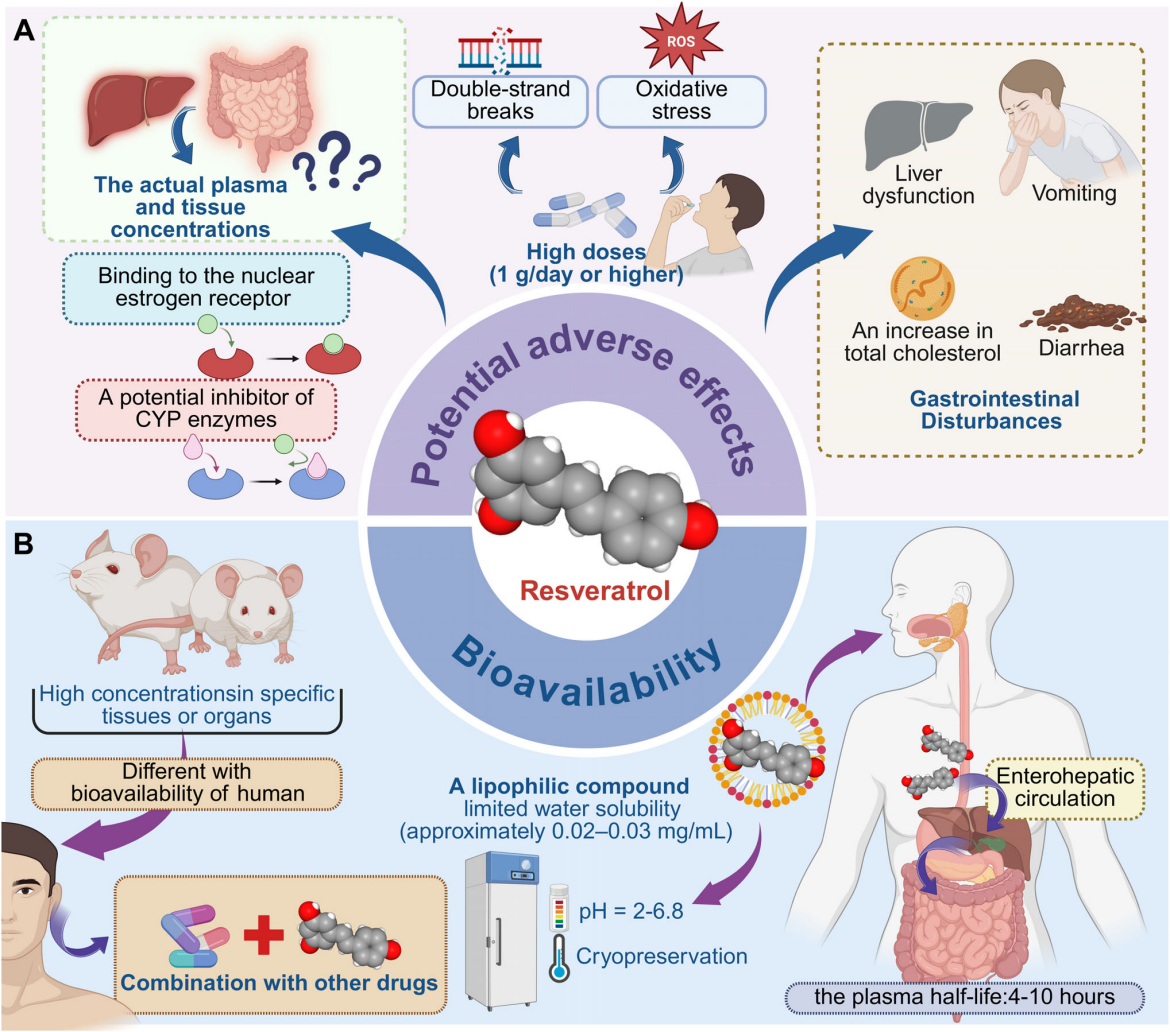
Potential adverse effects and bioavailability of RES. The potential adverse effects involved in RES mainly focus on the toxic effects caused by high doses. More attention should be paid to the research of RES metabolism and drug combination for its bioavailability. CYP, cytochrome P450; pH, hydrogen ion concentration; ROS, reactive oxygen species.

Currently, most clinical trials acknowledge the efficacy of RES in humans (Table 2). Additionally, the majority of studies have expanded beyond examining the singular effects of RES on diseases, instead exploring its potential as a supplement in combination with other drugs. This approach aims to either compensate for the limitations of standalone drug therapies or enhance the efficacy of existing treatments. As a result, the research and development of RES are no longer confined to a single mode of delivery. RES demonstrates promising therapeutic potential across a range of diseases, including cardiovascular conditions like atherosclerosis, cancer, neurodegeneration, and other degenerative disorders. Its efficacy is attributed to its ability to modulate multiple biological processes, such as regulating oxidative stress, apoptosis, autophagy, and inflammation. Additionally, RES acts as a hormone, influencing metabolic pathways and further contributing to its therapeutic effects. However, the number of RES studies involving human subjects remains limited. While the number of relevant clinical trials has steadily increased in recent years, the long‐term effects of RES remain uncertain, posing a significant barrier to its widespread clinical application. As a result, RES continues to be primarily regarded as a dietary supplement or nutraceutical. Additionally, the preservation of RES is another critical issue that requires attention. RES appears to be better preserved in an acidic environment (pH = 2–7) at room temperature, while it undergoes rapid hydrolysis in an alkaline environment, with significant hydrolysis beginning at pH levels above 6.8 [241]. Cryopreservation is a method employed to enhance the stability and preserve RES more effectively [242]. Given the crucial role of RES in slowing aging and preventing associated diseases, it is advisable to conduct further in vitro and in vivo studies focusing on delivery methods, dosage, and the long‐term effects of RES. These studies will help determine the optimal dose for various delivery methods. RES, when administered daily at doses of 2 g or less, does not seem to induce significant adverse effects (Figure 6).

**TABLE 2:** Clinical trials of RES.

Author	Year	Disease	Dosage	Effect	References
Battaglia	2022	Infertility	150 mg/d	RES can use mitomiRNAs to change granulosa cell transcripts and proteins, which will change the follicular microenvironment.	[243]
Kelly M Jardon	2024	Overweight/obesity	RES (80 mg/d)+ epigallocatechin‐3‐gallate (EGCG) (282 mg/d)	RES+ EGCG supplementation did not affect gut microbiota composition.	[244]
Christelle Nguyen	2024	Knee osteoarthritis	40 mg (2 caplets) twice a day for 1 week, then 20 mg (1 caplet) twice a day, 6 months in total.	Oral RES did not reduce pain in people with painful knee OA.	[245]
Colin J. Gimblet	2024	Chronic kidney disease and diabetes	400 mg/d	Endothelial function was enhanced by RES supplementation. RES enhanced the dilatation caused by flow.	[246]
Mitzi Marlotte van Andel	2024	Marfan syndrome (MFS)	500 mg/d	In adult patients with MFS, RES therapy for a year may stabilize the aortic development rate.	[247]
Graziamaria Corbi	2023	Bone loss	RES (25 mg) + equol (10 mg)	Both bone mineral density and bone turnover indicators are positively modulated by the combination.	[178]
Beatriz Isabel García‐Martínez	2023	Type 2 diabetes	1000 mg/d or 500 mg/d	The antioxidant effect of 1000 mg/d of RES is more effective than that of 500 mg/d.	[248]
Fernanda Navas Reis	2025	Dental caries	100 mg/d	RES dramatically decreased mutans streptococci and biofilm metabolic activity at 50 and 200 µg/mL, respectively. RES is a significant antibiotic and antibacterial.	[249]
Michał Ławiński	2025	Head and neck cancer	400 mg/d	The RES group showed a considerable increase in glutathione peroxidase (GPx), SOD, malondialdehyde (MDA), total antioxidant capacity (TAC), and phase angle.	[250]
Luana Almeida Gonzaga	2025	Coronary artery disease	RES (500 mg/d) + beetroot extract (500 mg/d)	The combination improved vagal regulation and heart rate recovery compared with rest.	[251]
Xuehui Zheng	2023	Hypertension	400 mg/d	RES supplementation can relieve left atrial remodeling, improve left ventricular diastolic function, and potentially alleviate cardiac fibrosis in hypertensive individuals.	[252]
Özge Erol Do˘ gan	2024	Ulcerative colitis	RES (500 mg/d) + mediterranean diet (MD)	The additional advantage might be minimal, and the main effect did not differ substantially from that of the MD alone.	[253]
Araceli Montoya‐Estrada	2024	Postmenopausal women with insulin resistance	RES (500 mg/d) + vitamin C	Supplementation with this combination of antioxidants significantly decreases markers of oxidative stress and TAC. Combined treatment can reduce protein damage more effectively than single treatment.	[254]

## 6 Conclusions

RES exhibits broad therapeutic potential in degenerative, cardiovascular, and NDs through modulation of multiple signaling pathways, offering antioxidant, anti‐inflammatory, and antiaging effects. While effective in improving joint health, cardiovascular function, and cancer outcomes, its clinical application is hindered by low bioavailability and dose‐dependent risks. Current evidence supports moderate doses (<2 g/day) as safe, but long‐term safety and optimized delivery systems require further exploration. Bridging the gap between preclinical promise and clinical application requires rigorous clinical trials and the development of innovative formulations to fully realize the therapeutic potential of RES.

## Author Contributions

Zhuo‐qun Ren: investigation, writing – original draft, writing – review and editing, and visualization. Sheng‐yuan Zheng: investigation, writing – original draft, writing – review and editing, and visualization. Zhengcheng Sun: investigation, writing – original draft, writing – review and editing, and visualization. Yan Luo: investigation, writing – original draft, writing – review and editing, and visualization. Yu‐tong Wang: writing – review and editing. Ping Yi: conceptualization, writing – review and editing. Yu‐sheng Li: conceptualization, writing – review and editing. Cheng Huang: conceptualization, writing – review and editing, and supervision. Wen‐feng Xiao: conceptualization, writing – review and editing, supervision. All authors have read and approved the final paper.

## Conflicts of Interest

The authors declare no conflicts of interest.

## Ethics Statement

The authors have nothing to report.

## Data Availability

The authors have nothing to report.
